# Supplementary material for: Evidence supported by Mendelian randomization: impact on inflammatory factors in knee osteoarthritis
Source: Front Med (Lausanne). 2024 May 28;11:1382836. doi: 10.3389/fmed.2024.1382836 (PMC11165061; doi:10.3389/fmed.2024.1382836)
Supplement: Supplementary file 1 [file Data_Sheet_1.ZIP › data/GCST90274823_5e6/823.leaveoneout.pdf]

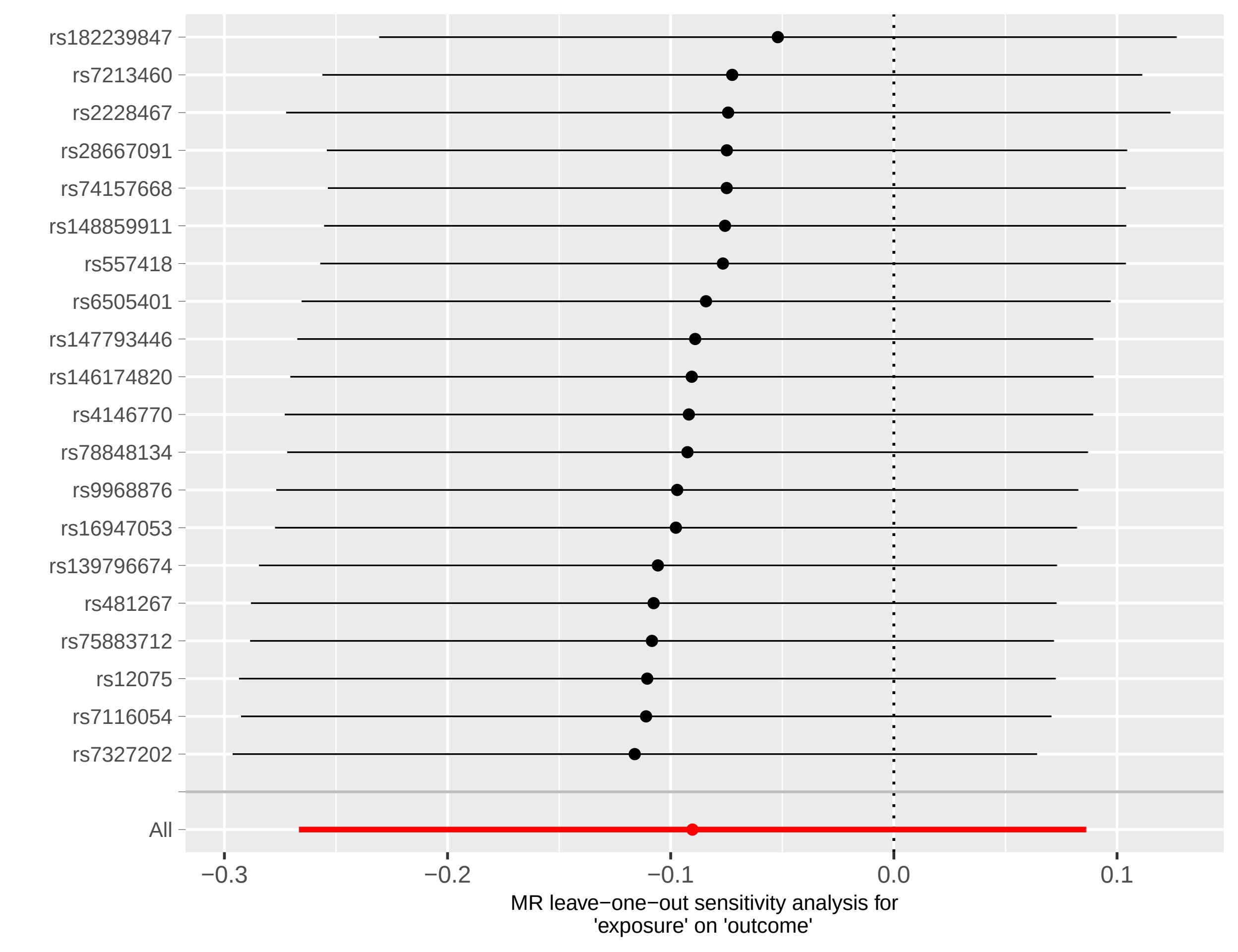
rs182239847
rs7213460
rs2228467
rs28667091
rs74157668
rs148859911
rs557418
rs6505401
rs147793446
rs146174820
rs4146770
rs78848134
rs9968876
rs16947053
rs139796674
rs481267
rs75883712
rs12075
rs7116054
rs7327202
All
−0.3
−0.2
−0.1
0.0
0.1
MR leave−one−out sensitivity analysis for
'exposure' on 'outcome'
